# A Pentaglot Dictionary of the Terms Employed in Anatomy. Physiology, Pathology, Practical Medicine, Surgery, Obstetrics, Medical Jurisprudence, Materia Medica, Pharmacy, Medical Zoology, Botany, Chemistry; in Two Parts: Part I—With the Leading Term in French, Followed by the Synonymes in the Greek, Latin, German, and English; Explanations in English; and Copious Illustrations in the Different Languages. Part II—A German-English-French Dictionary, Comprehending the Scientific German Terms of the Preceding Part

**Published:** 1845-07

**Authors:** 


Art. VII.—*A Pentaglot Dictionary of the Terms employed in Anatomy, Physiology, Pathology, Practical Medicine, Surgery, Obstetrics, Medical Jurisprudence, Materia Medica, Pharmacy, Medical Zoology, Botany, and Chemistry;* in Two Parts: Part I—*with the leading term in French, followed by the Synonymes in the Greek, Latin, German, and English; Explanations in English; and copious Illustrations in the different Languages.* Part II—*A German-English-French Dictionary, comprehending the Scientific German Terms of the preceding Part.* By Shirley Palmer m.d., of Tamworth and Birmingham.—*London,* 1845. 8vo, pp. 656.

In our Journal for October 1836, (Vol. II, p. 508,) we gave a very full and very favorable notice of the first two Parts of this work,—the plan and character of which are set forth in the title which we have transcribed in full. The long delay of the present Part has—we are sorry to see from the preface—been partly occasioned by the author's ill health, and has partly arisen from extrinsic causes for which he is not responsible. Notwithstanding the radical defect, of the French and not the English being made the leading language of the dictionary, and although the original plan, as we stated in our former notice, is faulty, both as to what is given and what is withheld, we do not hesitate to say that the whole is in the highest degree creditable to the author. It is indeed a splendid monument of his learning, industry, and talents, and cannot fail to be most useful to all who understand French. Indeed, the possession of it is indispensable to every medical man who looks beyond the limits of English medical literature. The German vocabulary appended to the present part renders the dictionary now equally available to the German as to the French reader; and if the author carries out his plan of compiling a Supplement containing similar vocabularies of the Latin, Italian, and English synonyms, the work will be then perfectly complete, and the inconvenience of the French vocabulary be entirely removed. We strongly recommend the learned lexicographer to lose no time in taking in hand this most important and almost necessary complement of his labours. We regard the whole medical profession throughout the world, as under great obligations to Dr. Palmer for what he has already done, and we earnestly recommend his work to all our readers at home and abroad.

